# A Meta-Analysis of the Impact of Anaerobic Soil Disinfestation on Pest Suppression and Yield of Horticultural Crops

**DOI:** 10.3389/fpls.2016.01254

**Published:** 2016-08-26

**Authors:** Utsala Shrestha, Robert M. Augé, David M. Butler

**Affiliations:** Department of Plant Sciences, University of Tennessee, KnoxvilleTN, USA

**Keywords:** anaerobic/biological soil disinfestation, meta-analysis, soil borne pathogens, nematodes, weeds, suppression, yield

## Abstract

Anaerobic soil disinfestation (ASD) is a proven but relatively new strategy to control soil borne pests of horticultural crops through anaerobic decomposition of organic soil amendments. The ASD technique has primarily been used to control soil borne pathogens; however, this technique has also shown potential to control plant parasitic nematodes and weeds. ASD can utilize a broad range of carbon (C) amendments and optimization may improve efficacy across environments. In this context, a meta-analysis using a random-effects model was conducted to determine effect sizes of the ASD effect on soil borne pathogens (533 studies), plant parasitic nematodes (91 studies), and weeds (88 studies) compared with unamended controls. Yield response to ASD was evaluated (123 studies) compared to unamended and fumigated controls. We also examined moderator variables for environmental conditions and amendments to explore the impact of these moderators on ASD effectiveness on pests and yield. Across all pathogen types with the exception of *Sclerotinia* spp., ASD studies show suppression of bacterial, oomycete and fungal pathogens (59 to 94%). Pathogen suppression was effective under all environmental conditions (50 to 94%) and amendment types (53 to 97%), except when amendments were applied at rates less than 0.3 kg m^-2^. The ASD effect ranged from 15 to 56% for nematode suppression and 32 to 81% for weed suppression, but these differences were not significant. Significant nematode moderators included study type, soil type, sampling depth, incubation period, and use of mixed amendments. Weed suppression due to ASD showed significant heterogeneity for all environmental conditions, confirming that these studies do not share a common effect size. Total crop yield was not reduced by ASD when compared to a fumigant control and yield was significantly higher (30%) compared to an unamended control, suggesting ASD as a feasible option to maintain yield without chemical soil fumigants. We conclude ASD is effective against soil borne pathogens and while not conclusive due to a limited number of studies, we expect the same for nematodes and weeds given observed effect sizes. Findings should assist researchers in exploring ASD efficacy in particular environmental conditions and allow for development of standard treatment protocols.

## Introduction

Methyl Bromide (MeBr), a broad-spectrum soil fumigant, was completely phased out in 2005 (with the exception of critical use exemptions) due to its stratospheric ozone depleting nature. Specialty crop growers have used this fumigant to control soil borne pathogens, nematodes, and weeds since the mid 20th century. Due to restriction on its use, growers are seeking alternatives that will provide comparable crop yield to that of MeBr. A number of chemical fumigant alternatives have been registered as replacements to MeBr fumigation (Rosskopf et al., 2014), but growers may not be willing or able to adopt them due to geographic limitations, reduced efficacy, safety issues, and regulatory constraints of these chemicals (Csinos et al., 2002; Martin, 2003). Further, worldwide awareness of environmental degradation and reduced-pesticide agriculture concepts (Carvalho, 2006) is driving many growers to seek non-chemical techniques to control crop pests. Non-chemical techniques such as flooding, solarization, steaming, and biofumigation (with cruciferous plant residues) are some available options for disease suppression. However, these generally environmentally friendly approaches have limitations (Shennan et al., 2010; Muramoto et al., 2014), such as high use of water (Runia and Molendijk, 2010; Runia et al., 2014a), high temperature requirements (Katan, 1981), use of costly equipment (Backstrom, 2002; Runia and Molendijk, 2010) and site-specific variability (Larkin and Griffin, 2007; Lopez-Aranda, 2014), respectively.

Another promising non-chemical option available to growers is anaerobic soil disinfestation (ASD), also known as biological soil disinfestation or anaerobically mediated biological soil disinfestation, which has been studied since 2000 in Japan (Shinmura, 2004; Momma, 2008), the Netherlands (Blok et al., 2000; Messiha et al., 2007) and the USA (Butler et al., 2012b; Rosskopf et al., 2014; Shennan et al., 2014). This technique relies on organic amendments to supply labile C to soil microbes to create anaerobic conditions in moist and plastic-covered soil. Soil microbes consume available oxygen and depletion of oxygen shifts the balance toward facultative anaerobes. Gasses (such as CO_2_, NH_3_, H_2_S, CH_4_, and N_2_O) and volatile fatty acids (VFAs) produced as a result of microbial decomposition of labile C during ASD lead to suppression of plant pathogens and nematodes. Among these compounds, VFAs (e.g., butyric acid and acetic acid), are particularly known to contribute to the soil disinfestation process (Momma et al., 2006).

Anaerobic soil disinfestation is an environmentally friendly pest control practice (Porter et al., 2010; Shennan et al., 2014; Rosskopf et al., 2015) where soil microbial growth can be enhanced, and soil fertility potentially enhanced by addition of organic amendments. A number of active research programs across the world continue to refine ASD techniques to control plant pathogens, nematodes, and weeds, and to further elucidate mechanisms of ASD treatment success (Shennan et al., 2014). Although ASD incurs relatively low implementation costs when locally available amendments are utilized, currently, ASD application in the USA has largely been limited to a few organic crop producers and early-adopter conventional growers. ASD requires further refinement of protocols to system variables and cost benefit analysis in comparison to other chemical fumigants (Butler et al., 2012b; Shennan et al., 2014). Quantitative review of ASD literature may be useful to researchers in terms of clarifying its efficacy across environments and help to make more exacting recommendations for wide-scale adoption.

Only narrative reviews of ASD amendments and ASD comparisons in different countries have been published (Shennan et al., 2014; Rosskopf et al., 2015; Strauss and Kluepfel, 2015). However, a quantitative synthesis of the literature in reference to the efficacy of ASD on a range of soil borne pathogens, nematodes and weeds has not been reported. Meta-analysis is a powerful tool that uses a set of statistical techniques to analyze independent studies quantitatively rather than qualitatively (Ojiambo and Scherm, 2006). The meta-analytic approach has provided useful results in medicine and psychology, and has been increasingly applied in agro-ecological systems and pest management (Madden and Paul, 2011; Ngugi et al., 2011; Poeplau and Don, 2015). The purpose of this meta-analytic review of previously published results on pest suppression due to ASD is to understand the efficacy of this non-chemical practice on a range of soil borne pathogens, nematodes and weeds. The meta-analysis also addresses comparative data on pathogens, nematodes and weeds using different moderator groups or explanatory variables. Likewise, ASD effectiveness on crop yield is an important study group for meta-analysis that can help growers to make ASD adoption decisions. Many researchers rely on results from lab tests or pot (e.g., greenhouse, growth chamber) studies only. However, soil disinfestation using organic amendments in field conditions is a challenge for researchers as pathogen suppression is subject to numerous environmental factors such as soil temperature, soil type, pathogen types, and more (Bonanomi et al., 2010). Moderator analysis is thus important to understand how these factors influence the efficacy of treatment. In this study, we examined the overall impact of various environmental and ASD treatment factors as moderators on ASD efficacy and effect size of pest suppression and yield.

## Materials and Methods

### Data Collection

**FIGURE 1:**
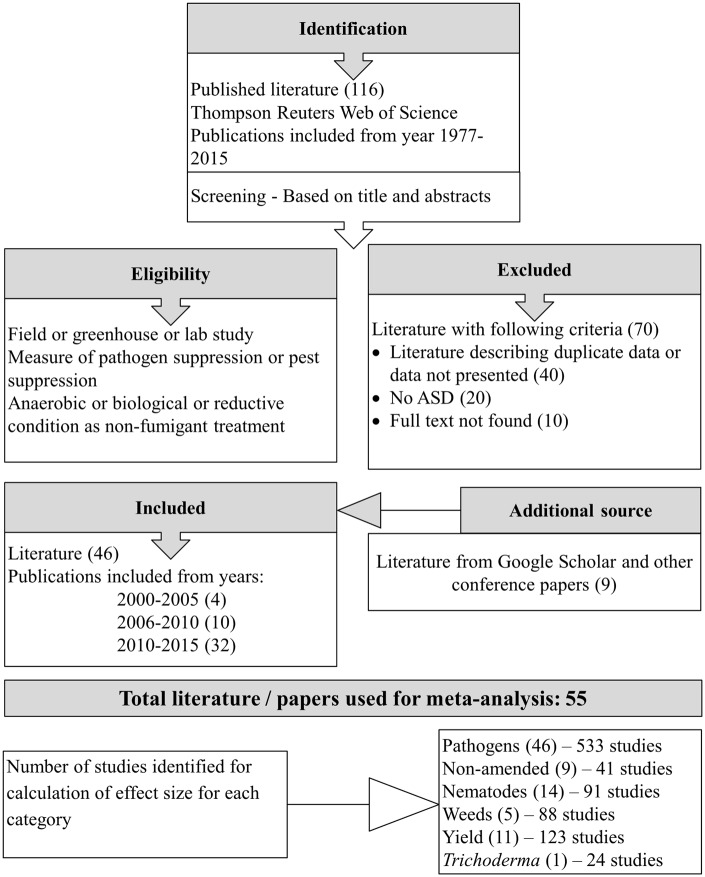
**Flow diagram showing the study selection procedure**.

Literature databases were explored using the search engine Thompson Reuters Web of Science on August 20, 2015. The terms used for the initial search, “soil disinfestation” OR “soil amend^∗^” OR “soil treat^∗^,” returned 78,019 search results. These search results were filtered to 116 articles using the search terms “ASD” OR “biological soil disinfestation” OR “reductive soil sterilization” OR “non-chemical fumig^∗^” OR “non-chemical alternative^∗^”. Records were retrieved from Web of Science Core Collection (70), CABI (37), BIOSIS Citation Index (6), and MEDLINE (3). Five books were excluded from 116 articles, and of the remaining 111 eligible articles, 65 were excluded because data described was presented in other original articles, full text could not be found, or did not meet one of the following inclusion criteria related to ASD-treatment: ASD treatment not applied, ASD was not compared with unamended control, or experiment was conducted in petri dishes only. In addition to the remaining 46 articles, we identified nine additional eligible articles using ‘Google scholar^TM^’ search. The meta-analysis included a total of 55 published and unpublished works (posters, theses, and conference papers) spanning 16 years from 2000 to 2015 and written in English (50), Japanese (2), Dutch (3), and Chinese languages (1) (**Figure 1**).

We collected treatment means and sample sizes from each study to evaluate effectiveness of ASD for pest suppression (soil borne pathogens/diseases, nematodes and weeds) and crop yield in relation to 11 factors identified as moderator variables. If the means were reported in graphical form, we used WebPlotDigitizer (Rohatgi, 2011) to estimate their values. ASD treatment means were those that used any type of C amendment(s), soil saturation or flooding and covering of soil (usually polyethylene mulch) during the study period, while the unamended and covered or non-covered treatments were considered control means. Only for yield response, we also collected means of fumigated treatments to compare with ASD treatment means. Multiple treatments or pathogens from one article were treated as independent studies (sometimes referred to as paired observations in the meta-analysis literature) and represented individual units in the meta-analyses. For example, (Butler et al., 2012b) reported pathogen data for two trials for seven different C amendments, resulting in 14 studies from that article. Although designating multiple studies from one publication has the disadvantage of increasing the dependence among studies that for the purposes of meta-analysis are assumed to be independent (Gurevitch and Hedges, 1999), the greater number of studies increases statistical power (Lajeunesse and Forbes, 2003). This approach has been used commonly in plant biology meta-analyses (e.g., Holmgren et al., 2012; Veresoglou et al., 2012; Mayerhofer et al., 2013; Chandrasekaran et al., 2014). The entire data set included 900 studies from eight countries (**Table 1**).

**Table 1:** Number of studies by country and USA states and response variables examined.

Serial number	Country	Soil borne pathogens^1^	Nematodes	Weeds	Yield	Non-amended	Trichoderma
(1)	Argentina	2	-	-	-	-	-
(2)	Belgium	-	2	-	-	-	-
(3)	China	56	-	-	1	4	-
(4)	UK	4	-	-	-	-	-
(5)	Japan	84	2	-	-	7	-
(6)	Netherlands	117	54	20	4	19	-
(7)	Sweden	12	-	-	-	8	-
(8)	USA (California)	36	-	3	56	-	-
(9)	USA (Florida)	111	28	25	32	-	24
(10)	USA (Tennessee)	91	-	40	30	-	-
(11)	USA (Washington)	20	5	-	-	3	-
	Grand total	533	91	88	123	41	24


*^1^Does not include studies without organic amendment (i.e., anaerobic and flooding only studies; 41 studies).*

### Moderator Variables

Several variables affecting pest suppression and yield were categorized and employed in moderator analysis. Our first moderator of interest was the method of characterizing ASD efficacy against each pest (i.e., ‘measure of efficacy moderator’), which represented studies that reported ASD effectiveness against pathogen, nematode and weed abundance in various quantifiable units (e.g., counts of pests, germination of pest propagules, ratings of disease; **Table 2A**). The different levels of ‘measure of efficacy’ were analyzed separately for each pest to understand the variation in effect sizes (**Figure 3**). We categorized soil borne pathogens into three levels: bacterial, fungal or oomycete and within each are specific pathogens (**Table 2B**). We also separated the beneficial soil organism *Trichoderma* to evaluate ASD effects. Further, realizing importance of the *Fusarium* genus that has been widely studied, we categorized *Fusarium* (*F*) spp. into six levels according to species and forma speciales (f. sp.) [*Fusarium* spp*., F. oxysporum* (*F. o.*), *F. oxysporum* f. sp. *asparagi, F. oxysporum* f. sp. *cubense, F. oxysporum* f. sp. *spinaciae, F. oxysporum* f. sp. *lycopersici*]. We also categorized available studies on nematodes and weeds according to their genus (**Tables 2C,D**). Yield had two levels, unamended control and fumigated control (**Table 2E**). We did not examine total vs. marketable yield as a moderator due to insufficient studies representing the total moderator level and we included total yield as a proxy for marketable yield where marketable yield was not reported. In addition, we recorded information for six categorical environmental moderators (explanatory variables) as study type, soil temperature, soil type, control type (with or without plastic mulch), depth of sampling, and incubation period for both pests and yield (summarized in **Table 2F**). These moderators are likely important determinants of the effectiveness of ASD in response to pest control and crop yield. In addition, ASD highly relies on amendments for C supplement and directly affect the ability of ASD to suppress pests. Accordingly, ASD amendment was categorized in four moderators: form (liquid or solid), single amendment or mixed, type, and rate (**Table 2G**). For environmental condition and amendment groups all moderator levels may or may not be present in the analysis.

**Table 2:** Levels and attributes within each categorical moderator variable tested for significance of pest suppression and yield responses

Categorical moderator variables	Levels	Attributes
**(A) Measure of efficacy (three levels)**	Pathogen	Colony size, germination (%), infection (%), colony forming units (log), microsclerotia count


	Nematode	Mass in root (g), hatching (%), counts, rating of disease


	Weed	Count, germination (%)


**(B) Soil borne pathogen genera (three levels)**	Bacterial (1)	*Ralstonia*


	Oomycete (2)	*Phytophthora, Pythium*


	Fungal (7)	*Cylindrocarpon, Fusarium, Macrophomina, Rhizoctonia, Sclerotium, Sclerotinia, Verticillium*


**(C) Nematodes (four levels)**	Plant parasitic	*Globodera, Pratylenchus, Meloidogyne* and others *(Heterodera, Pratylenchus, Trichodorus, Tylenchorhynchus*)


**(D) Weeds (five levels)**	Weed type	*Amaranthus retroflexus, Chenopodium album*, *Cyperus esculentus, Digitaria sanguinalis*, and others


**(E) Yield (two levels)**	Control	Fumigated control, unamended control


**(F) Environmental conditions**


(i) Study type(Two levels)	Small scale	Study mostly in controlled environment using glass, bag, bucket, box, pot, growth chamber


	Large scale	Field/plots


(ii) Soil temperature (Three levels)	Low	<16°C


	Moderate	16 to 35°C


	High	>35°C


(iii) Soil type (Six levels)	Sandy	Sandy, sandy peat, sandy loam, loamy sand, sandy clay loam, glacial sand


	Clay	Clay, clay loam


	Loam	Loam, silty loam, marine loam


	Gray lowland	Poorly drained soil


	Volcanic ash	Andosol


	Other media	Greenhouse soil, peat, perlite, and other


(iv) Control (two levels)	Yes	Plastic sealed to create anaerobic conditions


	No	Uncovered treatment


(v) Depth of sampling (Three levels)	Shallow	0 to 5 cm


	Moderate	6 to 15 cm


	Deep	>15 cm


(vi) Incubation period	Variable	Ranged from <3 to >10 weeks


**(G) Amendments**


(i) Amendments form (two levels)	Liquid	Ethanol, organic acids, semi-solid molasses
	Solid	All other amendment types
(ii) Amendments mixed (two levels)	No	Single amendment only
	Yes	2 or >2 different amendments mixed
(iii) Amendment type (11 levels)	Agricultural by-product	Wheat bran, rice bran/straw, maize stalks/straw, molasses (solid and liquid), grape pomace, onion waste, potato residue
	Cruciferous	Arugula, broccoli, radish, mustard and other mustard products
	Combination	>2 amendments used
	Protein by-product	“Herbie^1^,” volatiles from “Herbie”
	Legume	Cowpea, crimson clover, hairy vetch, sunn hemp, alfalfa
	Grass	Oat, cereal rye, perennial ryegrass, Italian ryegrass, pearl millet, sorghum-sudangrass, wheat and other grasses
	Manure	Poultry litter with or without solarization, composted cattle manure
	Organic acid	Acetic acid, butyric acid, lactic acid, ‘SPK’
	Ethanol	Ethanol, bio-ethanol (0.5, 1, and 2%)
	Other C source	Glucose, sucrose, xylose, C media (other organic material)
(iv) Non-amended	No amendments	Anaerobic or flooding
(v) Rate per m^2^	Variable	Ranged from <0.3 to>9 kg


*^1^Proprietary blend of plant products, see Runia et al. (2014b) for more information.*

### Effect Size and Meta-Analysis

Our analyses followed the methodology and terminology of Borenstein et al. (2009) and were guided by the criteria suggested by Koricheva and Gurevitch (2014). We computed summary effects and associated statistics using Comprehensive Meta-Analysis Version 3 (CMA) software (Biostat, Englewood, NJ, USA; 2014). We used a random-effects model for the meta-analyses, considering that true effects are likely to have varied across studies (rather than a fixed-model, which assumes the same value or true effect for all studies).

The effect sizes were calculated as the natural log response ratio (*lnR*) of treatment mean to control mean and subjected to analysis of overall effect sizes (pest suppression and yield responses) of ASD for each moderator. *lnR* for each observation was calculated as

$$InR=In\left(X_{t}/X_{c}\right)$$

where *X*_t_ is the ASD treatment mean and *X*_c_ is the control mean (unamended, untreated or fumigated control mean for yield). The log transformation was needed to balance positive and negative treatment effects and to maintain symmetry in the analysis (Borenstein et al., 2009). Given that approximately 80% of papers did not report a measure of dispersion, non-parametric variance was calculated as:

$$V_{InR}=(n_{t}+n_{c})/\left(n_{t}*n_{c}\right)$$

where, *V_ln_*_R_ is the variance of the natural log of the response ratio, and *n*_t_ and *n*_c_ are the sample sizes of the treatment and control means, respectively. In studies in which several treatments were compared with one control group, sample size of the one control group was partitioned across treatment means. For example, for a study with one control and three treatments, each having four replicates, the control sample size (4) was divided by three. This was done to avoid overweighting studies by incorporating the same experimental units (e.g., plot, plants) in more than one effect size. Values of zero are biologically common but mathematically not possible to incorporate into meta-analysis (ratio denominator cannot be 0; cannot calculate the natural log of 0). A common technique used in the medical literature is to add a small fixed number to any zero value (NCSS Statistical Software, 2015). In pathogen control research, however, this technique yields very inconsistent results, owing to the wide variety of units and the wide range of maximal pathogen growth/survival values. Further, small non-zero values result in unreasonably inflated response ratios. In order to analyze effect sizes of zero and near zero, we calculated 1% of the highest pathogen abundance value for a study and raised any other value below 1% to that level: for example, to 0.75 for 75 log CFU g^-1^ of soil, and to 0.03 for 3.0 cm colony diameter. Negative values of pathogen abundance were equated to zero before applying the 1% adjustment.

Heterogeneity was assessed with the *Q* statistic, a measure of weighted squared deviations. Total heterogeneity (Q_t_) is composed of expected or within-study variation (Q_w_) and excess or between-study variation (Q_b_). Heterogeneity was quantified using *I*^2^, a descriptive index that estimates the ratio of true variation (heterogeneity) to total variation across studies:

$$I^{2}=(Q_{t}-df)/Q_{t}*100\%$$

where df denotes the expected variation Q_w_ and Q_t_ - df the excess variation (Q_b_) *I*^2^ is set to 0 when df exceeds Q_t_. A value of 0% indicates no true heterogeneity, and positive values indicate true heterogeneity in the data set with larger values reflecting a larger proportion of the observed variation due to true heterogeneity among studies. Assumptions of homogeneity were considered invalid when *p*-values for the *Q* test (*P*_hetero_) for heterogeneity were less than 0.1 (e.g., Bristow et al., 2013; Iacovelli et al., 2014). We assumed a common among-study variance across moderator subgroups.

### Publication Bias and Sensitivity Analysis

Publication bias is the term applied to a body of research in the refereed literature that is systematically unrepresentative of all completed studies (Rothstein et al., 2006). Literature reviews can be subject to publication bias, and the standard narrative review more so than quantitative meta-analysis review (Borenstein et al., 2009). The issue is raised more often with meta-analysis, likely because this method is intended to be comprehensive. The concern is the possibility that significant treatment differences are more likely to be published than non-significant findings. Direct evidence of publication bias is difficult to obtain, but it is important to check for it (Sutton, 2005; Madden and Paul, 2011; Koricheva and Gurevitch, 2014). Methods generally involve exploring the relationship between study effect size and precision. The idea is that studies with smaller sample sizes or higher variance will tend to have larger effect sizes than larger studies with greater precision. Hence, potential publication bias was assessed statistically with Begg and Mazumbar rank (Kendall) correlation and represented graphically with funnel plots of effect sizes versus their standard errors (estimated from their non-parametric variances; Begg and Mazumdar, 1994; Borenstein, 2005; Borenstein and Cooper, 2009; Borenstein et al., 2009). Duval and Tweedie (2000) iterative trim and fill method was used to demonstrate how the summary effect size would shift if apparent bias were to be removed. Sensitivity analysis was performed for the overall summary effects by removing one study and re-running the meta-analysis for every study in the analysis. This shows how much each study contributed to the summary effect, by noting how much the summary effect changes in its absence. Possible temporal changes in effect size were evaluated with meta-regression using publication year as a quantitative moderator (Koricheva and Gurevitch, 2014). Meta-regression analysis was conducted with the CMA software, with the restricted maximum likelihood and Knapp-Hartung methods (IntHout et al., 2014).

## Results

We did not see evidence of publication bias. Visually, the funnel plots for each of the summary effects showed no pattern that would reflect bias toward not reporting small positive or negative effect sizes (**Table 3**). Large and small studies across the range of standard errors had the expected variability around the summary effect size. Within the Begg and Mazumdar (1994) rank correlation test, each of the summary effects had absolute Kendall tau values below 0.02, indicating no publication bias (no tendency for effect sizes to increase as study size decreases; **Table 3**). The Duval and Tweedie (2000) trim and fill procedure imputes missing studies needed to make the funnel plot symmetrical, removing the most extreme small studies and recomputing the effect size at each iteration until the funnel plot is symmetric on either side of the new (adjusted) summary effect. To maintain proper variance, the original studies are added back into the analysis along with a mirror image for each. The adjusted value is suggestive only, as when between-study heterogeneity exists (as was the case in our meta-analysis), trim and fill may inappropriately adjust for publication bias where none exists and thereby led to spurious changes in the summary effect (e.g., Terrin et al., 2003). A main concern about missing studies is that their absence in the analysis may result in an exaggerated summary effect. In our analysis, however, the summary value adjusted for potential missing studies is further from zero than the original value for the pathogen and weed summary effects (**Table 3**). The test revealed no potential missing studies and hence no adjustments for nematode control or yield assessed relative to unamended controls or to fumigated controls. Therefore, the trim and fill analysis indicates no concern that publication bias has resulted in inflated summary effects. In fact, if the suggested adjustments are legitimate for pathogen and weed control (if there really are missing studies) then the Duval and Tweedie analysis points to an even greater impact of ASD in controlling these pests.

**Table 3:** Measures used in characterizing publication bias for each effect size (after Borenstein, 2005).

Effect sizes	Summary effect^1^				Funnel plot^2^	Kendall tau^3^	Duval and Tweedie adjusted^4^	No. impute^5^
					
	*n*	*lnR*	*p*	No. var.				
Pathogen	533	-1.12	<0.001	0.005	No	-0.07	-1.29	66
Nematode	91	-0.04	0.027	0.060	No	-0.14	-0.04	0
Weed	88	-0.75	0.002	0.058	No	-0.11	-1.49	17
Yield with unamended control	68	0.26	0.034	0.015	No	0.02	0.26	0
Yield with fumigated control	55	0.05	0.687	0.018	No	-0.07	0.05	0


*^1^Summary effect: n, number of studies; ln*R*, natural log of overall summary effect; *p*, probability that summary effect ≠ 0; No. var., number of different variance values of studies comprising the summary effect. ^2^Funnel plot appears asymmetrical. ^3^Begg and Mazumdar Kendall rank correlation: tau = rank correlation coefficient (with continuity correction). ^4^Duval and Tweedie trim and fill: adjusted summary effect after imputing missing studies using an iterative trim and fill procedure. ^5^No. impute, number of studies imputed in the trim and fill exercise.*

The stability of the overall summary effects was assessed with sensitivity analysis. One study was removed and the summary effect recalculated, and this was repeated for all studies to determine how much any one study affected the summary effect size. The study with the largest influence on pathogen control was study 379 (*lnR* = -5.511, *Verticillium* treatment, Runia et al., 2014b), whose removal changed the summary effect by 0.4% (from 67.5 to 67.1% reduction in pathogens). The study with the largest influence on nematode control was study 720 [*lnR* = -0.401, sandy soil with solid amendment treatment, van Overbeek et al. (2014), whose removal changed the summary effect by 3.4% (from 36.4 to 33.0% reduction)]. The study with the largest influence on weed control was study 794 (*lnR* = -0.810, trial 5, McCarty, 2012), whose removal increased the size of the summary effect by 2.4% (from 52.7 to 55.1% reduction in weeds). The study with the largest influence on yield was study 871 [*lnR* = 0.205, eggplant treatment, (Butler et al., 2012b), whose removal reduced the summary effect by 5.9% (from 28.6 to 22.7% promotion of yield, relative to unamended controls)] (**Supplementary Table 1**).

Koricheva and Gurevitch (2014) recommended testing whether a summary effect has changed over time, when studies comprising the effect have been published over many years. Changes in the summary effect could potentially result from publication bias, changes in methodology, or real biological changes. Investigating chronology (year of publication), as a quantitative moderator using meta-regression, ASD control of pathogens has changed slightly over time; the yearly average change was -1.0% (*p* = 0.81) over the data’s 16 publication years. ASD control of nematodes has changed somewhat more over its 12 years of data, with an average decline of -1.8% per year (*p* = 0.07). There was an insufficient range of publication years of articles and studies to characterize the influence of ASD on weed control or yield.

For our analysis, a natural log response ratio (*lnR*) value below zero indicates suppression of pests (i.e., soil borne pathogens, plant parasitic nematodes and weeds), a value above zero indicates an increase in pests with ASD, and a zero value signifies no effect of ASD treatments on pest suppression. The levels within moderators are considered significantly different from each other or from the overall mean when confidence intervals do not overlap. Heterogeneity (the presence of underlying structure, i.e., true differences among studies) within moderators was characterized by *I*^2^ and *P*_hetero_. For each pest, we grouped our results as each pest type or crop type, experimental condition and amendment used in ASD. We reported ASD yield response separately for the fumigated control and unamended control.

### Measure of Efficacy

**FIGURE 2:**
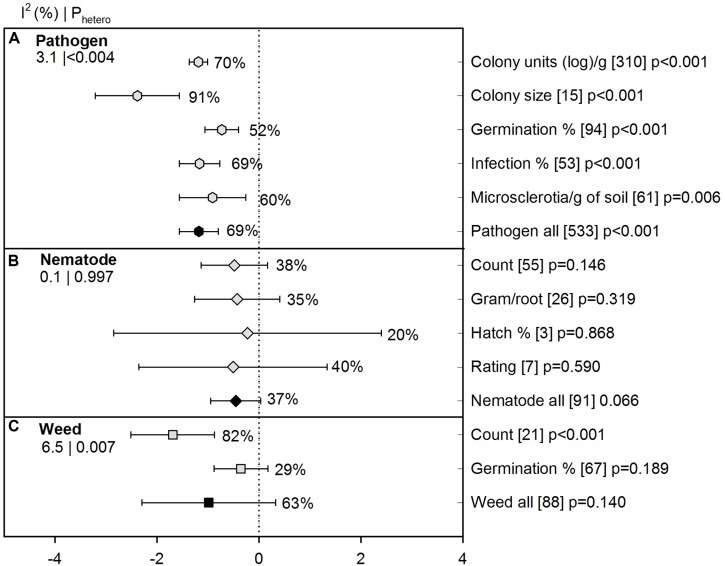
**Weighted summary effect sizes (*lnR*) and 95% confidence intervals (CIs) for ASD moderator ‘measure of efficacy’ (various measures of pathogen growth and survival used in the literature).** Comparisons among levels of **(A)** Pathogen (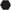-hexagon symbols), **(B)** Nematode (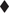-diamond symbols), and **(C)** Weed (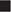-square symbols). For each level of moderator, values to the right of the CI line with negative effective size are percent pest suppression and with positive effect size are percent of promotion. Number of studies reporting data for each level of moderator is given in brackets. The moderator level was significantly different from zero if *p*-value ≤ 0.05. Values below panel titles to the left are *I*^2^ (percentage of heterogeneity due to true variation among moderator levels) and *P*_hetero_ (test of the null hypothesis, that all studies share a common effect size if *P*_hetero_ > 0.1) for each moderator. Open symbols denote levels of each moderator (subgroups); closed symbols denote overall moderator summary effect.

We detected an overall negative ASD effect on pathogen abundance in various quantifiable units (-1.18 [CI -1.56 to -0.80]). When growth of pathogens was measured in colony size, ASD effect was highest with 91% suppression and was significantly different from other units (**Figure 2A**). Such a high significance in colony size was reported as a pathogen suppression indicator during ASD treatment in one article (Mazzola and Hewavitharana, 2014) with 15 studies, but realizing the importance of the study and the slight difference in the overall effect size (5%) after removal of the colony size unit, we decided to include all studies in our analysis. In the case of nematodes, all units ranged between 20 and 40% and we observed 37% overall effectiveness for nematode suppression (**Figure 2B**). Number of weeds in terms of ‘count’ (i.e., population or density) was highly reduced by ASD compared to germination of weed propagules (82% vs. 29%; **Figure 2C**).

### Pathogens

**FIGURE 3:**
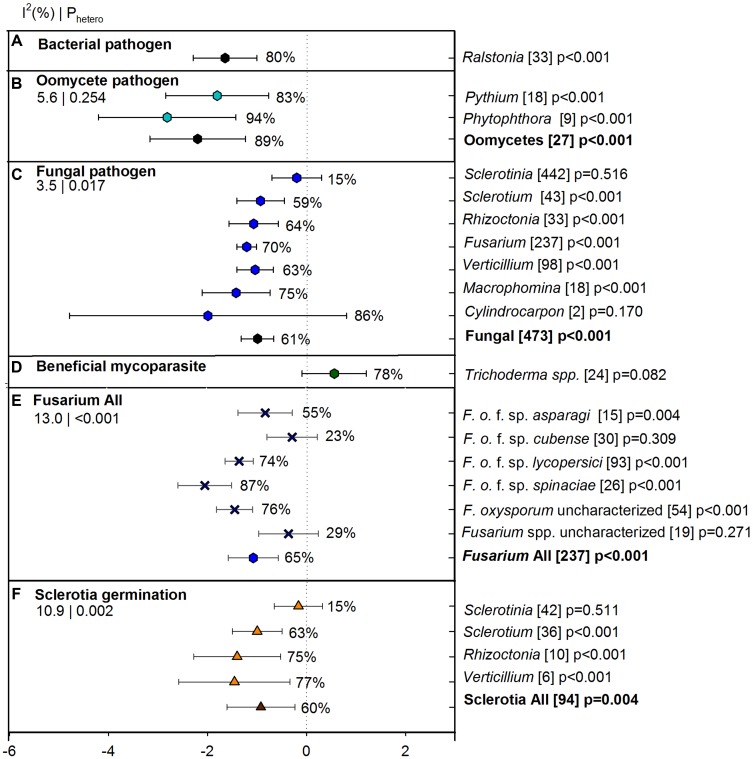
**Weighted summary effect sizes (*lnR*) and 95% CIs for ASD effect on suppression of pathogens and beneficial mycoparasites.** Comparisons among levels of **(A)** bacterial pathogen, **(B)** oomycete pathogen, **(C)** fungal pathogen, **(D)** beneficial mycoparasite, **(E)** *Fusarium* all, and **(F)** sclerotial germination. For each level of moderator, values to the right of the CI line with negative effective size are percent pathogen suppression and with positive effect size are percent of promotion. Number of studies reporting data for each level of moderator is given in parentheses. The moderator level was significantly different from zero if *p*-value ≤ 0.05. Values below panel titles to the left are *I*^2^ (percentage of heterogeneity due to true variation among moderator levels) and *P*_hetero_ (test of the null hypothesis, that all studies share a common effect size if *P*_hetero_ > 0.1) for each moderator.

Overall ASD effect on suppression of different soil borne pathogens which were categorized as bacterial, oomycete or fungal pathogens was -1.22 [CI -1.57 to -0.87] showing 70% suppression over 533 studies (**Figure 3**). Suppression was significantly higher for oomycete pathogens than for fungal pathogens and similar for oomycete and bacterial pathogens (**Figure 3A**). Between oomycetes, *Phytophthora* had higher suppression by ASD than *Pythium* but the difference was not statistically significant as CIs for the two summary effects overlapped (**Figure 3B**). More studies on ASD were conducted for fungal pathogens (seven soil borne genera), among which *Sclerotinia* was least suppressed by ASD (15%). The ASD effect on *Sclerotinia* suppression significantly differed from *Fusarium* suppression (70%). All soil borne pathogens except *Sclerotium* were better suppressed by ASD (>63%) than unamended controls although these pathogens did not differ significantly (**Figure 3C**). *Cylindrocarpon* was the most suppressed pathogen (86%), but with high CI values. To get an idea of the ASD effect on beneficial organisms, we also evaluated the ASD effect on *Trichoderma* (*n* = 24) and we observed a positive effect of ASD on these beneficial fungi (**Figure 3D**).

Since *Fusarium* was the most studied pathogen with 237 individual studies, it was of interest to observe the ASD effect on different host specific *Fusarium* pathogens (f. sp.) within *Fusarium* level. It also included uncharacterized *F. oxysporum* (54) and uncharacterized *Fusarium* spp. (19). The overall effect size of *Fusarium* level within pathogen was -1.05, [CI -1.55, -0.54] (representing an ASD suppression of 65% in raw terms), with significant heterogeneity *p* < 0.001. True variation among studies, estimated by *I*^2^, accounted for 13% of total variation. We observed a significantly higher suppression level of ASD for the spinach and tomato wilt pathogens; *F. oxysporum* f. sp. *spinaciae* (87%) and *F. oxysporum* f.sp. *lycopersici* (74%), respectively. The uncharacterized *F. oxysporum* also showed a similar effect size and was significantly higher than other levels of *Fusarium* (76%). The *F. oxysporum* f. sp. *cubense* and other uncharacterized *Fusarium* spp. were less suppressed by ASD (**Figure 3E**). When we compared the ASD effect on sclerotial germination percentage of sclerotia-bearing pathogens, we found germination percentage was effectively lowered in *Verticillium*, *Rhizoctonia*, and *Sclerotium*, but not in *Sclerotinia* (**Figure 3F**).

#### Experimental Conditions for Pathogen Studies

**FIGURE 4:**
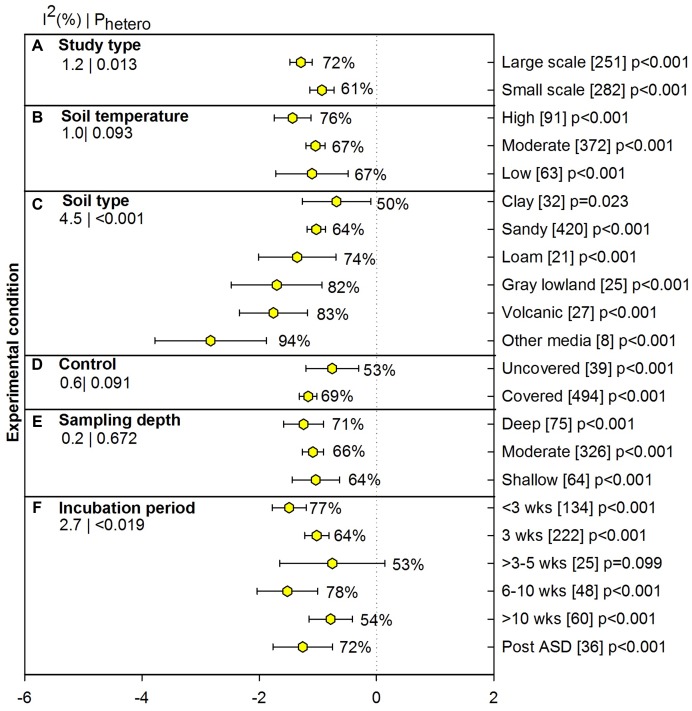
**Weighted summary effect sizes (*lnR*) and 95% CIs for ASD effect on pathogen suppression under various experimental conditions.** Comparisons among levels of **(A)** study type, **(B)** soil temperature, **(C)** soil type, **(D)** control, **(E)** sampling depth, and **(F)** incubation period. For each level of moderator, values to the right of the CI line with negative effective size are percent pathogen suppression and with positive effect size are percent of promotion. Number of studies reporting data for each level of moderator is given in parentheses. The moderator level was significantly different from zero if *p*-value ≤ 0.05. Values below panel titles to the left are *I*^2^ (percentage of heterogeneity due to true variation among moderator levels) and *P*_hetero_ (test of the null hypothesis, that all studies share a common effect size if *P*_hetero_ > 0.1) for each moderator.

Experimental conditions for pathogens included meta-analysis results from only soil borne pathogens and excluding beneficial mycoparasites and non-amended treatments (e.g., flooding only). Small studies carried out in the laboratory and growth chamber conditions showed 61% pathogen suppression and large studies conducted in the field and the greenhouse showed slightly higher suppression (72%, **Figure 4A**). At high soil temperature, the pathogen reduction by ASD effect was ∼10% higher than at moderate and lower soil temperatures (**Figure 4B**), however, a significance difference was not observed due to extended confidence interval of high temperature. The ASD treatment in volcanic soil from Japan showed significantly higher suppression of pathogens than sandy soil (83% vs. 64%), while neither type of soil differed from clay, gray low land and loam soil. ASD effectiveness was significantly higher for ‘other media,’ which included greenhouse media, perlites, etc. (94%; **Figure 4C**). Pathogen suppression was not affected by whether ASD treatments involved covering (**Figure 4D**), and degree of suppression has been similar across different sampling depths (64 to 71%; **Figure 4E**). ASD incubation periods of greater than 10 weeks and 3 to 5 weeks were less effective than other periods. It is interesting to see >78% pathogen suppression for the less than a 3-week period. Three weeks is by far the most used ASD incubation period for pathogen suppression (222 studies) and is among the most effective periods (64%; **Figure 4F**).

#### Amendment Effect on Pathogen Suppression

**FIGURE 5:**
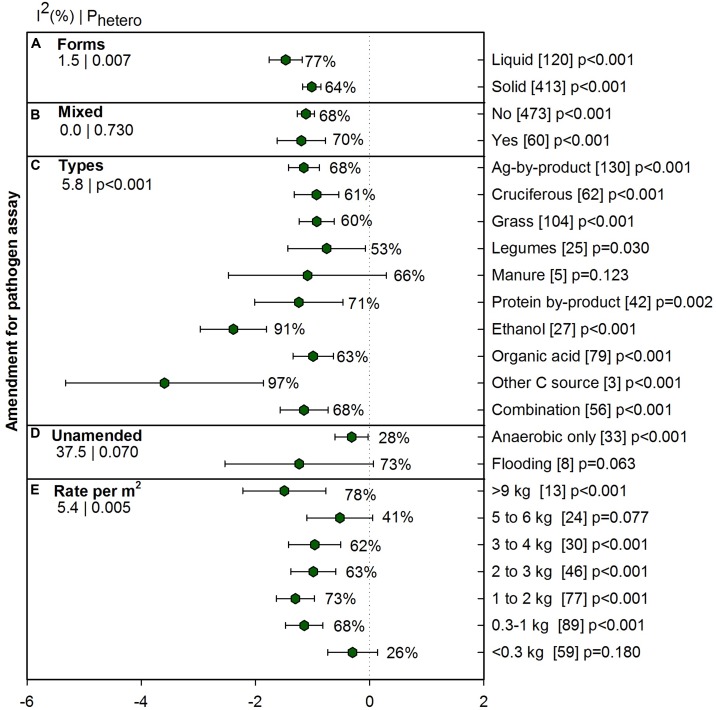
**Weighted summary effect sizes (*lnR*) and 95% CIs for ASD amendment effect on pathogen suppression.** Comparisons among levels of **(A)** forms, **(B)** mixed, **(C)** types, **(D)** unamended and **(E)** Rate per m^2^. For each level of moderator, values to the right of the CI line with negative effective size are percent pathogen suppression and with positive effect size are percent of promotion. Number of studies reporting data for each level of moderator is given in parentheses. The moderator level was significantly different from zero if *p*-value ≤ 0.05. Values below panel titles to the left are *I*^2^ (percentage of heterogeneity due to true variation among moderator levels) and *P*_hetero_ (test of the null hypothesis, that all studies share a common effect size if *P*_hetero_ > 0.1) for each moderator.

The type and amount of amendment is a crucial component of ASD to provide labile C to microbes, and so we examined amendment characteristics for influence on the efficacy of ASD on pathogen suppression. Across all pathogen studies (*n* = 533) five amendment moderators were categorized and analyzed separately. **Figure 5A** provides results of liquid vs. solid amendments (*n* = 533) and **Figure 5B** depicts mixed vs. non-mixed amendments (*n* = 533). We found 533 studies were amended with various C sources (**Figure 5C**) and 41 studies were unamended and were analyzed separately (**Figure 5E**). Ethanol, organic acid and other C source (glucose, sucrose, and xylose) in amendment type moderator are applied as liquid amendments. Besides liquid molasses included in ag-by-product, all other amendments were solid amendments.

Amendment in liquid form was more effective than solid form, 77% vs. 64% (**Figure 5A**). Mixing different amendment types did not increase the effectiveness of ASD as compared to single amendment (**Figure 5B**). Most C amendments significantly reduced pathogen measures (**Figure 5C**) and overall ASD effect on plant pathogens was -1.24, (CI [-1.56, -0.91] *p* < 0.001). When ASD was conducted with ethanol, ASD effectiveness increased dramatically and was significantly different from other amendments: organic acid, combination, ag-by-product, cruciferous, grass and legume (91%). ‘Other C source,’ which includes glucose, sucrose, and xylose showed the most pathogen suppression among amendments. Suppression of pathogens was, however, lower than 61% when amendments were cruciferous, legume and grass. We also examined anaerobic and flooding situations (i.e., without C amendment) to gain a sense of whether pathogen survival under these conditions was similar to ASD treatment and we found that while flooding was effective, anaerobic conditions are not as effective as ASD (28%, **Figure 5D**). Effectiveness of ASD on pathogen suppression also relies on rate of amendments. Amendment rates less than 0.3 kg m^-2^ and 5 to 6 kg m^-2^ did not show as much suppression as other rates (**Figure 5E**). Generally, the trend was that higher suppression was observed with higher rates of amendment but in meta-analysis of amendment rate, we could see response of pathogen suppression is not only subject to application rate. Detailed analysis of pathogen suppression with various amendment types under different conditions are presented in **Supplementary Table 2**.

### Nematode Suppression

**FIGURE 6:**
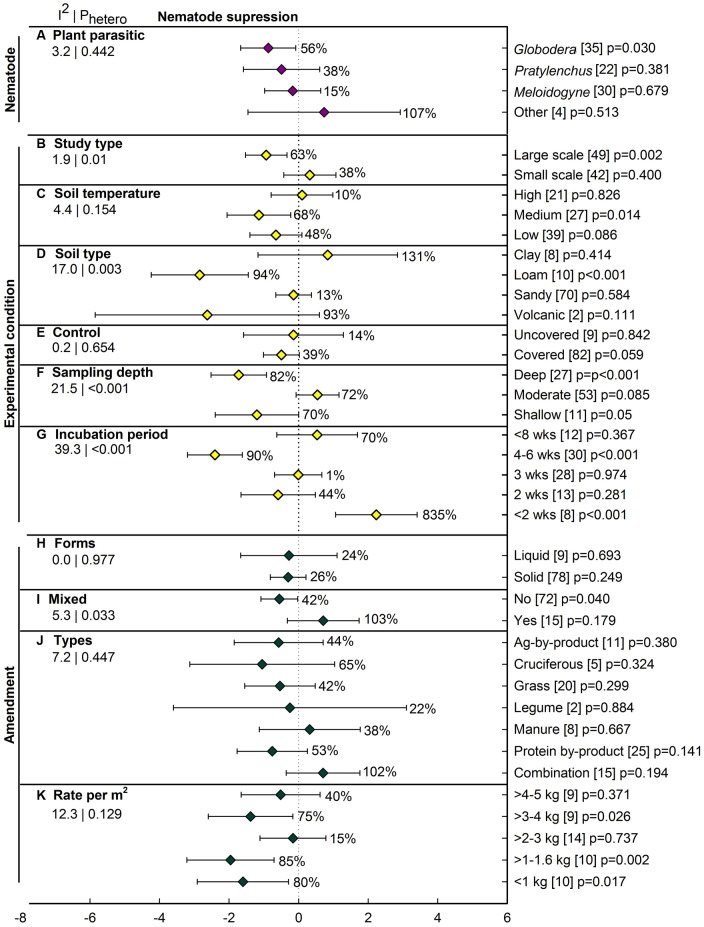
**Weighted summary effect sizes (*lnR*) and 95% CIs for ASD effect on nematode suppression.** Comparisons among levels of **(A)** plant parasitic nematode (Other = *Heterodera*, *Trichodorus*, and *Tylenchorhynchus*), **(B)** study type, **(C)** soil temperature, **(D)** soil type, **(E)** control, **(F)** sampling depth, **(G)** incubation period, **(H)** forms, **(I)** mixed, **(J)** types, and **(K)** Rate per m^2^. For each level of moderators, values to the right of the CI line indicate percent changes induced by ASD in raw terms: negative values represent suppression or reduction, positive values represent promotion. Number of studies reporting data for each level of moderator is given in parentheses. The moderator level was considered significantly different from zero if its *p*-value ≤ 0.05. Values below panel titles to the left are *I*^2^ (percentage of heterogeneity due to true variation among moderator levels) and *P*_hetero_ (test of the null hypothesis, that all studies share a common effect size if *P*_hetero_ > 0.1) for each moderator.

Over all studies, ASD decreased nematode abundance by 37% (*lnR* = -0.45), with the confidence interval slightly overlapping zero (*p* = 0.066; **Figure 2B**). The four individual efficacy measures ranged from 20 to 40%, with confidence intervals also crossing zero. Among the three most studied plant parasitic genera, ASD-induced inhibition was significant only for *Globodera*, at 56% (**Figure 6A**). The summary effect was not significant for *Pratylenchus*, *Meloidogyne* and the three genera grouped as ‘Other.’ Among the six moderators characterizing experimental conditions, most have at least one level with a significant ASD effect (**Figures 6B–G**). Unlike pathogen suppression, ASD has resulted in substantial nematode suppression in large-scale studies (63%, *p* = 0.002), with no suppression in small-scale studies (38%, *p* = 0.40) (**Figure 6B**). Suppression was greatest at moderate soil temperatures (68%, *p* = 0.01) and insignificant at the higher and lower reported temperatures (**Figure 6C**). The ASD effect varied with soil type, with significant suppression of nematodes (94%) occurring only in loam soils (**Figure 6D**). The size of the ASD-induced suppression has not differed as a function of its comparison to uncovered vs. covered controls (**Figure 6E**). Sampling depth markedly affected estimation of ASD efficacy, with nematodes reduced by 82 and 70%, respectively, in deep and shallow regions of the soil profile while at moderate depth a near significant ASD stimulation of nematodes has been observed (**Figure 6F**). Incubation of less than 2 weeks has dramatically promoted nematode survival, while an incubation of 4–6 weeks has resulted in significant nematode suppression (**Figure 6G**). Amendment characteristics have had less influence on the extent to which ASD suppressed nematode than fungal pathogens (**Figures 6H–K**). Liquid and solid forms of amendment have given similar nematode control (**Figure 6H**). Not mixing amendments has been far more efficacious than mixing them (**Figure 6I**). None of the amendment types resulted in a significant effect of ASD on nematode suppression (**Figure 6J**), although the small numbers of studies representing several of the amendment types give low statistical power for resolving differences. It was surprising that ASD showed nematode suppression at amendment rates less than 2 kg m^-2^ and 3 to 4 kg m^-2^ but rates at 2 to 3 kg m^-2^ and 4 to 5 kg m^-2^ did not show any significant effect (**Figure 6K**), but again, the relatively low number of studies which were performed under varying amendment types and soil temperatures limits interpretation.

### Weed Suppression

**FIGURE 7:**
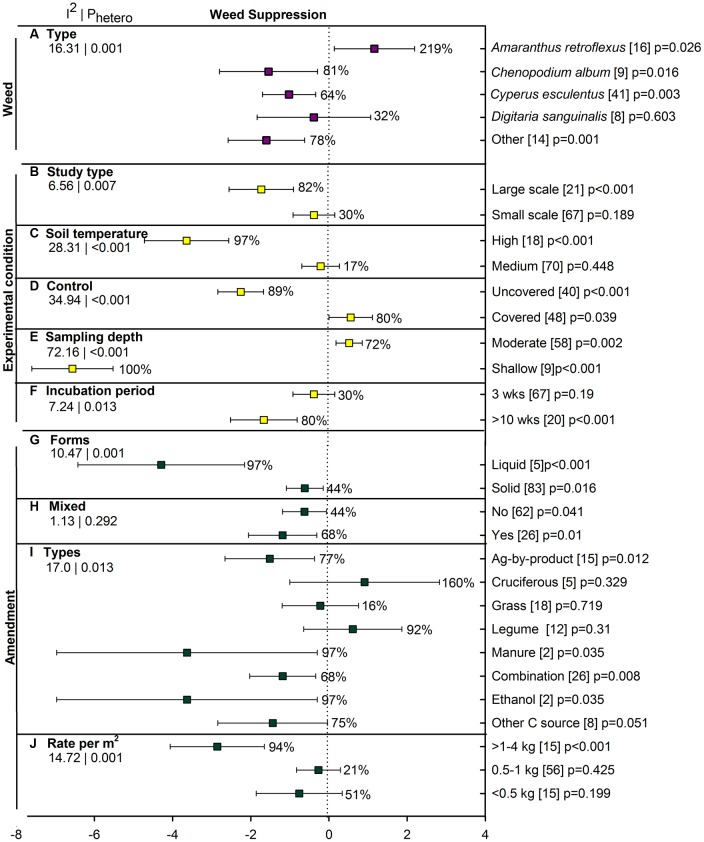
**Weighted summary effect sizes (*lnR*) and 95% CIs for ASD effect on weed suppression.** Comparisons among levels of **(A)** weed type, **(B)** study type, **(C)** soil temperature, **(D)** control, **(E)** sampling depth, **(F)** incubation period, **(G)** forms, **(H)** mixed, **(I)** types, and **(J)** rate per m^2^. For each level of moderator, values to the right of the CI line with negative effective size are percent weed suppression and with positive effect size are percent of promotion. Number of studies reporting data for each level of moderator is given in parentheses. The moderator level was significantly different from zero if *p*-value ≤ 0.05. Values below panel titles to the left are *I*^2^ (percentage of heterogeneity due to true variation among moderator levels) and *P*_hetero_ (test of the null hypothesis, that all studies share a common effect size if *P*_hetero_ > 0.1) for each moderator.

Few studies have addressed the influence of ASD on weed suppression (88 studies from five publications) and all studies were conducted in sandy soil. Overall weed reduction was 63% when examined as both weed count and germination percentage (**Figure 2C**). Weed measures have been much more affected by ASD when assessed as weed population density (82%, *p* < 0.001) than as germination of introduced propagules (29%, *p* = 0.189). *Chenopodium album*, *Cyperus esculentus* (yellow nutsedge) and less frequently studied species have shown significant reductions with ASD (**Figure 7A**). *Digitaria sanguinalis* (crabgrass) has not been affected by ASD in the few studies reported, and growth of *Amaranthus retroflexus* (pigweed) has actually been substantially promoted by ASD. Large-scale application of ASD has resulted in significant weed suppression whereas small-scale application has not suppressed weeds (**Figure 7B**). The effect of ASD has been evident only when soil temperatures are high (**Figure 7C**). ASD treatments have suppressed weeds only when compared to uncovered controls; covering soils during treatment has given better weed control than ASD treatments (**Figure 7D**). Another interesting observation for ASD was seen for sampling depth (or burial depth), with shallow depths being significantly more suppressive to weeds and moderate depths promoting weed populations (**Figure 7E**). Incubation periods of greater than 10 weeks showed better control than a 3-week period, with the latter having little effect on weed measures (**Figure 7F**).

Each of the four amendment moderators affected ASD efficacy on weed suppression (**Figures 7G–J**). The applied liquid form showed 97% weed suppression, about twice as effective as solid amendments at 44% weed reduction (**Figure 7G**). Mixed and single amendment forms of ASD have had similar, significant effects (**Figure 7H**). Among amendment types, ag by-products, manure, ethanol and the less frequently used other C sources led to substantial ASD-induced weed suppression in the range of 77 to 97% (**Figure 7I**). ASD resulted in significant weed suppression only when the rate of amendment was greater than 1 kg m^-2^ (**Figure 7J**).

### Yield

**FIGURE 8:**
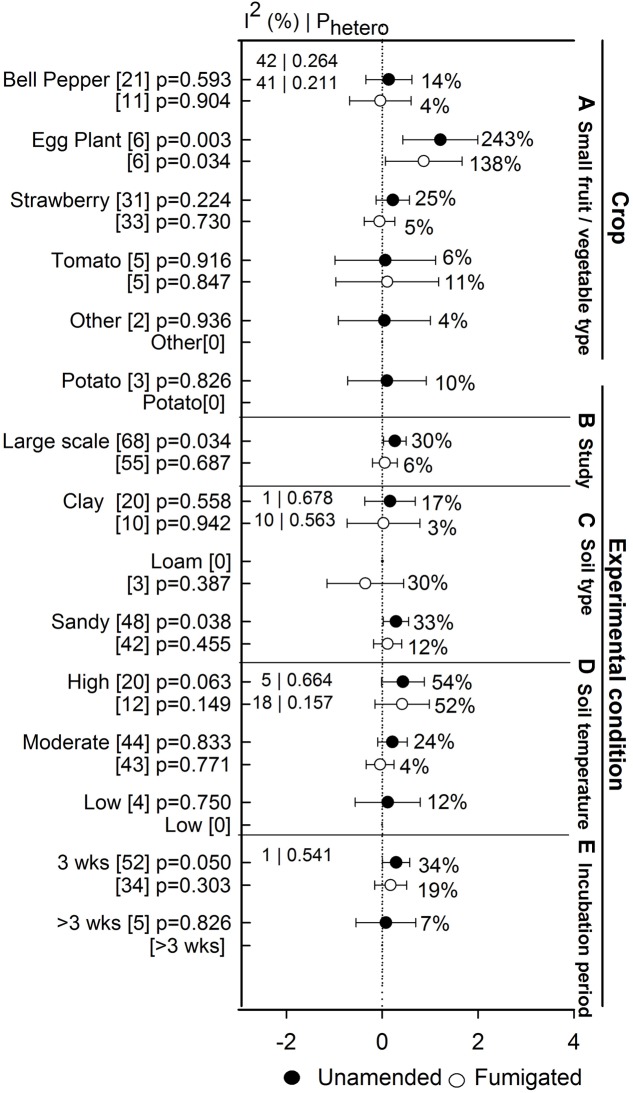
**Weighted summary effect sizes (*lnR*) and 95% CIs for ASD effect on yield response.** Comparisons among levels of **(A)** crop type, **(B)** study type, **(C)** soil type, **(D)** soil temperature, and **(E)** incubation period. For each level of moderator, values to the right of the CI line with negative effective size are percent yield decrease and with positive effect size are percent of yield increment. Number of studies reporting data for each level of moderator is given in parentheses. The moderator level was significantly different from zero if *p*-value ≤ 0.05. Values below panel titles to the left are *I*^2^ (percentage of heterogeneity due to true variation among moderator levels) and *P*_hetero_ (test of the null hypothesis, that all studies share a common effect size if *P*_hetero_ > 0.1) for each moderator. Closed symbols (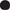) denote ASD compared with unamended untreated control; open symbols denote (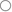) ASD compared with fumigated control.

**FIGURE 9:**
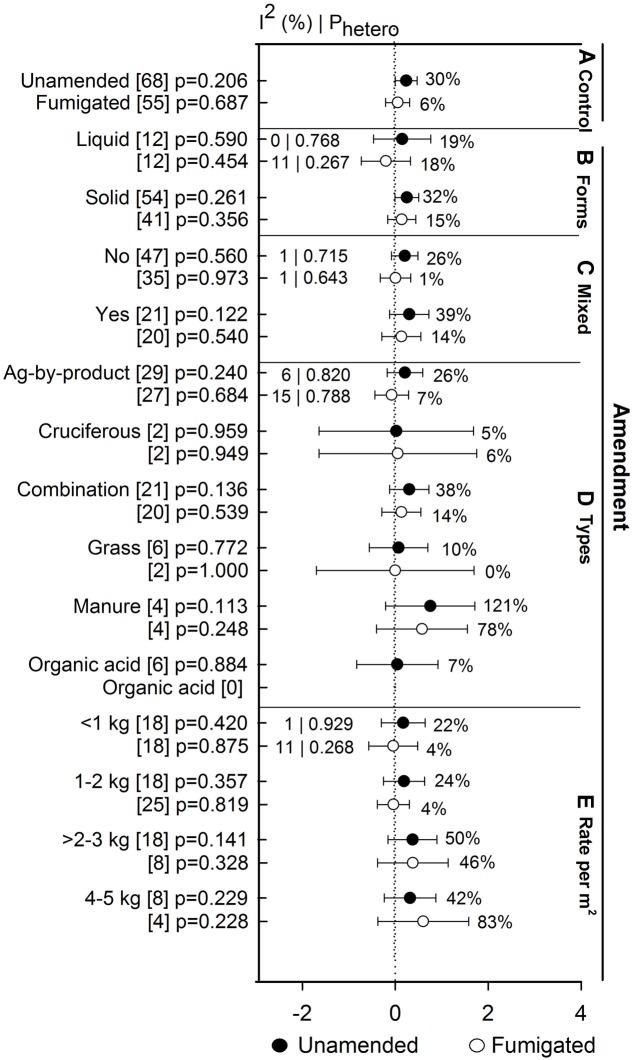
**Weighted summary effect sizes (*lnR*) and 95% CIs for ASD amendment effect on yield response.** Comparisons among levels of **(A)** control, **(B)** forms, **(C)** mixed, **(D)** types, and **(E)** rate per m^2^. For each level of moderator, values to the right of the CI line with negative effective size are percent yield decrease and with positive effect size are percent of yield increment. Number of studies reporting data for each level of moderator is given in parentheses. The moderator level was significantly different from zero if *p*-value ≤ 0.05. Values below panel titles are *I*^2^ (percentage of heterogeneity due to true variation among moderator levels) and *P*_hetero_ (test of the null hypothesis, that all studies share a common effect size if *P*_hetero_ > 0.1) for each moderator. Closed symbols (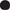) denote ASD compared with unamended untreated control; open symbols (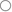) denote ASD compared with fumigated control.

Anaerobic soil disinfestation treatment promoted yields of eggplant when compared to both unamended and fumigated controls (>130%, **Figure 8A**). Yield of bell pepper, strawberry, tomato, potato, and other crops has remained unaffected by ASD. Within these crops, the lack of effect on yield occurs whether ASD efficacy is viewed relative to unamended or fumigated controls (**Figure 8A**). The absence of ASD influence on yield has not been affected by study type (**Figure 8B**). ASD tended to promote yield at sandy soil (33%, **Figure 8C**), higher temperatures (>54%, **Figure 8D**) and shorter incubation times (34%, **Figure 8E**). Yield response increased to 6% when ASD was compared with fumigated treatments and 30% with unamended control (**Figure 9A**). ASD effect on yield compared to both control treatments was highest for solid amendments compared to liquid (15 to 32%, **Figure 9B**). Mixing of amendments increased yield 13 to 14% in both cases (**Figure 9C**). Similar to weed suppression, manure amendment tended to have the most positive effect on yield in both cases (>78%, **Figure 9D**). In addition, yield response increased with respect to increase in application rate of amendment (**Figure 9E**).

## Discussion

### Is ASD Effective for Pathogen Suppression?

Our results indicate strong evidence of pathogen suppression by ASD and that ASD plays a critical role in minimizing pathogen inoculum by inhibiting germination of inoculum or reducing the vigor of pathogens. We observed that colony size as a ‘measure of efficacy’ of pathogen suppression was highly sensitive to ASD. Colony size during ASD would likely be affected by the range of volatile compounds and other toxic anaerobic decomposition by-products. Along with colony size, we also observed suppression of pathogens in terms of colony forming units, germination percentage, infection percentage and microsclerotia production. Given the various efficacy measurements, we confirmed that overwintering forms of pathogens that impact crops could potentially be effectively suppressed by ASD.

Studies have shown that ASD is effective in suppressing various soil borne pathogens (as reviewed by Shennan et al., 2014; Rosskopf et al., 2015) and our meta-analysis results were consistent with those narrative reviews. Our meta-analysis also demonstrated the importance of statistical power in terms of study number; for example, the only two studies for *Cylindrocarpon* (infection percentage) showed no statistical difference, although disease reduction was 86%. We are not surprised that banana wilt caused by *F. oxysporum* f. sp. *cubense* in China (Huang et al., 2015; Wen et al., 2015) was less suppressed by ASD than all other *Fusarium* spp., as use of amendments like rice and corn straw in these two reports, in conjunction with flooding of soil, likely have differing microbial responses compared to more labile C amendments. We observed a significantly higher suppression level of ASD for the spinach wilt pathogen *F. oxysporum* f. sp. *spinaciae* (87%) and the tomato wilt pathogen *F. oxysporum* f. sp. *lycopersici* (74%). For *Sclerotinia*, which was less affected by ASD, data were reported only from species *sclerotiorum* and it was reported that sclerotial germination was highly influenced by the low amendment rate and soil temperature (Butler et al., 2014b). Further, sclerotial viability, release of biochemical compounds, and infection ability vary under different growing conditions and ineffectiveness of ASD in such cases may relate to a combination of factors. At the same time, Thaning and Gerhardson (2001) reported sclerotia of *Sclerotium cepivorum* from onion was unaffected by ASD (since data were not reported, *S. cepivorum* is not included in the meta-analysis). On the other hand, sclerotia of *Verticillium* and *Sclerotinia* both failed to survive in the same study. Variability in sclerotial infection mechanisms (e.g., production of apothecia or mycelium; Imolehin et al., 1980) can also impact ASD effectiveness. Nevertheless, from our meta-analysis, we can grasp the degree of fungistasis (soil property preventing germination of viable propagules) being enhanced in ASD relative to size of sclerotia; specifically, compared to *Sclerotinia*, smaller sclerotia of other sclerotial pathogens are more effectively suppressed by ASD (see **Figure 3F**). Sclerotial germination is typically not reported in *Macrophomina*, as the size of sclerotia are too small to enumerate (100–200 um). Recent studies on the bacterial pathogen *Agrobacterium tumefaciens* in tree crops was reported to be suppressed by ASD (Strauss et al., 2014), confirming ASD can be expanded to target other new plant pathogens and other crops.

Interestingly, our meta-analysis showed that ASD promoted the population of the mycoparasite *Trichoderma*. This mycoparasite along with other fungi parasitizing sclerotia of *S. rolfsii* were reported in Thaning and Gerhardson (2001) and Shrestha et al. (2013). Likewise, occurrence of the *Sclerotinia sclerotiorum* sclerotial parasite *Coniothyrium minitans* was reported by Thaning and Gerhardson (2001). However, ASD effects on these beneficial organisms are not reported. Looking at the positive impact of ASD on *Trichoderma*, although non-significant in this study, suggests that more studies on ASD effects on beneficial microorganisms are needed. Studies have revealed that Firmicutes, Clostridia, and *Bacillus* are prominent in microbial communities during ASD (Mowlick et al., 2012). Further studies will help to further elucidate dynamics of beneficial organisms during and post-ASD treatments, which will allow for treatment adaptations to increase impact on beneficial organisms.

### Conditions Favoring ASD Effectiveness on Pathogen Suppression

Our analysis suggests that ASD can work as a replacement to chemical fumigants for pathogen suppression as we observed consistent pathogen suppression under various conditions (**Figure 4**). These results also suggest that ASD significantly suppresses pathogens across a range of temperatures. ASD treatments were more effective under higher soil temperature for both nurseries and field conditions. If soil temperature is relatively high (>16°C) the incubation period can be reduced to less than 3 weeks since our analysis showed >80% of pathogen suppression is achieved when temperature ranged from 16 to 30°C and pathogens were not suppressed (40%) when temperature was low (data not shown). However, under low temperature (<16°C), ASD can be effective when certain factors are modified, for e.g., *Ralstonia* and *Verticillium* under low temperature were effectively suppressed when higher amendment rates (grass) and longer incubation periods of 10 to 25 weeks were practiced.

It is not uncommon to see greater suppression of pathogens in media such as potting soil and other laboratory media other than soil, potentially due to reduced heterogeneity and reduced populations of other soil microorganisms than in field conditions. These media based studies are usually accompanied by smaller studies in a greenhouse, growth chamber, or laboratory with controlled environmental conditions. Among various types of soil, clay and sandy soils showed low suppression of pathogens in response to ASD treatment. Reasons for this observation may include low availability of C to microorganisms due to rapid loss of soluble C in sandy soil and greater adsorption and reduced water infiltration rate that affects the distribution of decomposition by-products in clay soils. Clay soils are also likely to be more buffered against changes in soil pH that may affect the accumulation of VFAs. Further, these acids are weakly adsorbed to the soil’s exchange phase and have rapid turnover rate with short half-life (Jones et al., 2003) and transitory when exposed from anaerobic to aerobic condition (Lazarovits et al., 2005). Whereas volcanic ash, loam and gray lowland soil showed more suppression than clay and sand as these soils are themselves more fertile with high mineral contents which often enhance microbial activity.

One of the benefits of ASD is that it may be able to control pathogens under relatively short incubation periods for a biological soil treatment. Surprisingly, ASD suppressed pathogens under relatively short incubation periods. For an incubation period <3 weeks, we noticed 77% pathogen control which was directly related to study type and soil type. Most of the studies with < 3-week incubation periods were reported from small-scale studies, including lab studies and other C sources with eight amendment types in this analysis (110 studies) and only 24 studies reported from large-scale studies, which included volcanic ash and gray lowland studies. Lower percentages of pathogen suppression for 3–5 weeks and >10 weeks incubation periods may be attributed to few amendment types (ag-by-product, brassica, grass or protein-by-product) included in the meta-analysis. Pathogen suppression even after ASD treatment (post ASD) duration reveals that ASD prevents resurgence of pathogens. However, post ASD treatments in this analysis included only organic acid and the C amendment in this case may create a different response than other amendments.

### Contribution of ASD Amendments to Pathogen Suppression

Amendments such as ethanol, organic acids and liquid molasses are easier to apply in the soil through drip application or by spraying. Liquid amendments are easily incorporated in soil and rapidly translocate to the soil profile, which our results suggest makes them more effective in ASD than solid amendments. In Japan, ethanol for ASD is already practiced at a relatively large scale (Momma et al., 2013) and in Florida liquid molasses is commonly used (Butler et al., 2012a; Rosskopf et al., 2014).

The categorization of amendment types in **Figure 5C** as moderator levels clearly shows differences in various C amendments for pathogen suppression. It also indicates the importance of moderator analysis as we get a clearer indication of effect sizes for various amendments. The category ‘other C sources’ in this analysis (glucose, xylose, and sucrose) showed the highest suppression of pathogens, and studies were conducted in plastic boxes against *Fusarium* pathogens. This illustrates that ASD is highly effective in controlled environments, likely due to high anaerobic activity and confinement of VFAs and other volatile compounds (Hewavitharana et al., 2014). Recently, Daugovish et al. (2015) used diluted glycerol as liquid amendment in field soil and found that this C source was not as effective as rice bran to create long lasting anaerobic conditions, which suggests that ASD effectiveness may in some cases differ in field conditions.

From our analysis, ethanol is established as the most effective ASD amendment in controlling plant pathogens. ASD effectiveness due to ethanol is directly related to concentration and incubation period (Momma et al., 2006); a minimum incubation of 9 days is required for effective ASD treatment when 0.5% (of soil volume) of ethanol is used. In addition, almost all amendments used as C sources in the studies in this meta-analysis are considered to produce high VFAs relative to unamended controls (**Figure 5**).

For effective disease suppression, relatively high rates of amendment incorporation are reported as necessary (Mowlick et al., 2013; Butler et al., 2014b). From our results, we confirmed higher amendment rates lead to higher suppression. However, amendment rates at 5–6 kg m^-2^ rate showed slightly less suppression and the reason may be that represented studies utilized only grass and cruciferous plants. These amendments are less readily decomposed due to more complex C compounds in whole plant tissue than in simpler and more labile C sources such as ethanol, molasses, and glucose. Our results do suggest that ASD implementation costs could potentially be lowered by application of low amendment rates in some cases (∼ 300 g m^-2^) of amendment, which should be studied further.

### ASD Effect on Nematode Suppression

Measure of efficacy results indicated that hatching and number of nematodes, infection incidence, and density of nematodes in roots were not significantly suppressed by ASD treatment. Only potato cyst nematode (*Globodera*) was effectively controlled by ASD and half of studies used protein-by product amendment (Runia et al., 2014b; Streminska et al., 2014; van Overbeek et al., 2014). *Meloidogyne* and *Pratylenchus* showed some suppression, but this was non-significant. Nematode studies were approximately seven times fewer than pathogen studies and **Figure 6** shows how this low number of studies affected nematode suppression evaluation, with large confidence intervals due to error (Borenstein et al., 2009). We observed that nematode suppression with ASD is not as effective as pathogen suppression. However, higher suppression of nematodes by ASD treatments in field conditions, high organic content soil (e.g., loam and volcanic soil) and 2 to 6 weeks of incubation period was observed. Both liquid and solid amendments seem effective in nematode control. Besides manure and combination levels, all other amendments applied in ASD suppressed nematodes. In our analysis, moderator levels manure and combination consist of poultry litter (seven studies each), which is known to have nematicidal activity (Riegel and Noe, 2000) and was always associated with soil solarization to increase soil temperatures. However, it was not effective enough for nematode suppression. Since the early twentieth century, studies have revealed that decomposed organic matter helps in reduction of nematodes (Linford et al., 1938). Reviews on various amendments and mechanisms of suppression against various nematodes are reported (Rodriguez-Kabana, 1986; Rodriguez-Kabana et al., 1987; Oka et al., 2007) but very few studies have been conducted to evaluate efficacy of ASD on nematode suppression. More studies are encouraged under a range of ASD treatment factors and environmental conditions in order to better evaluate ASD impact on plant parasitic nematodes.

### ASD Effect on Weed Suppression

Although there are few reports on weed suppression by ASD compared to pathogens and nematodes, our meta-analysis indicated that ASD is effective in suppressing weeds as well. Except *A. retroflexus*, all other weeds evaluated were found to be suppressed with ASD treatment (**Figure 7B**). *Amaranthus* is a troublesome persistent weed with an extended germination period (Karimmojeni et al., 2014) and the study included in our meta-analysis was a pot observation thus emphasizing the need for additional research. *Digitaria* suppression likely needs some refinement in ASD while *Cyperus* tuber germination was suppressed by ASD. Although these weed suppression studies were conducted in pots, we believe that ASD can be equally effective if used in field conditions as *C. album* and other weeds in a field study showed high suppression when grass and other C sources were used as amendments. An ASD effect on weeds at shallow depths with almost 100% control of weeds could potentially be of large benefit; however, this represents few observations (*n* = 9) which were reported from a single paper (Muramoto et al., 2008) conducted in pots, with high temperature and without a covered control. More studies are needed with more variables for such cases to better assess suppression effects. When amendments were in liquid form, almost 99% weed control was achieved and reasons are likely similar to that discussed previously for pathogen suppression. It was not surprising to see that ethanol and manure amendments in ASD are more capable of weed suppression than other amendments as these may promote more toxicity than other C sources to control the weed propagules. However, there is a need to explore more cover crops, ethanol and manures as ASD amendments, and for an increase in the number of these studies. For effective weed suppression, rates of amendments greater than 1 kg m^-2^ are likely needed.

### ASD Effect on Crop Yield

We found that total fruit yield of crops was not reduced by ASD when compared to a fumigant control and yield was significantly higher when compared to an unamended control. Our results indicate that ASD is promising for sandy soil and high soil temperature and the result may be due to suppression of pathogens and weeds by lethal temperatures, as well as substantial beneficial effects of organic matter additions on chemical, biological and physical properties of sandy soils (Butler et al., 2014a). Application of manures and increased amendment rate increased the yield (>50%) compared to both fumigated and unamended controls. However, due to low number of studies, we see overlapping of confidence intervals and it is expected that if the number of studies on ASD using manures increases, we may see a significant crop yield result from meta-analysis.

Not surprisingly, a far higher number of publications on ASD are related to disease suppression than to yield response. The small numbers of published yield studies do not allow a comprehensive meta-analysis. This, and the numerous variations inherent to field studies, led to large CIs and likely insufficient power to determine with statistical confidence if yield summary effects differ from zero. Further, analysis of yield data faces several limitations. First, many papers do not report standard deviation and so use of non-parametric variance may have added additional uncertainty to our results. Second, although our mean yields include mostly marketable yield, in some instances (20 studies from McCarty et al., 2014) we included total yield as a proxy for marketable yield where marketable yield was not reported. As concluded by Belova et al. (2013), the lack of detail provided in many studies about field experimental protocols, horticultural practices and field management history hinders conclusive analysis. The wide confidence intervals for yield in our results likely reflect the fact that yield is affected by many environmental factors, soil factors and other cultural practices.

## Conclusion

Given that pests evaluated in ASD studies differ widely in biological characteristics, it is not surprising that biologically based ASD treatments may differentially impact survival and growth of these organisms. ASD treatment showed a high reduction in bacterial (*Ralstonia*), oomycete (*Pythium* and *Phytophthora*) and fungal (except for *Sclerotinia*) pathogen inoculum. Among fungal pathogens, ASD response to pathogen supression was high for *Cylindrocarpon, Macrophomina*, *Fusarium*, *Rhizoctonia*, *Sclerotium*, and *Verticillium*. Among different host specific *F. oxysporum* pathogens, *F. oxysporum* f. sp. *spinaciae* and *F. oxysporum* f. sp. *lycopersici* were significantly suppressed by ASD. Under most environmental conditions (i.e., a range of study types, soil temperature, soil types and incubation period), suppression of pathogen inoculum due to ASD treatment ranged from 50 to 94%. While our results indicate that ASD is effective for suppression of a broad range of plant pathogens as compared to an unamended control across a range of amendment types, amendment rates (>0.3 kg m^2^), soil temperatures, soil types, and treatment incubation periods, research and demonstration studies often report variable results when compared to conventional soil fumigant controls. While this is not surprising given that ASD treatment relies on a more complex biological process that is influenced by environmental conditions and interactions with existing soil biology as compared to chemical fumigants, it does suggest that further refinement to improve ASD techniques could lead to more consistent field suppression compared to fumigants. Accordingly, ASD methods likely will need refinement based on the pests of interest and environmental conditions in a given production system. Due to a limited number of studies and variability in reported research, we cannot conclude that ASD is consistently effective in suppressing nematode or weed pests, although suppression has been achieved for some species under specific environmental and treatment conditions. Given broad-based suppression of plant pathogens under ASD treatments, future research should focus on further improving consistency of ASD treatment for soil borne plant pathogens to improve competitiveness of this biologically based technique with conventional soil fumigants.

## Author Contributions

US took the lead role in study design, data collection, and data analysis; and drafted the first version of the manuscript. RA and DB assisted with study design, data collection and analysis, and critically revised the manuscript.

## Conflict of Interest Statement

The authors declare that the research was conducted in the absence of any commercial or financial relationships that could be construed as a potential conflict of interest.
